# Supplementary material for: New Insights into Hemopexin-Binding to Hemin and Hemoglobin
Source: Int J Mol Sci. 2022 Mar 30;23(7):3789. doi: 10.3390/ijms23073789 (PMC8998376; doi:10.3390/ijms23073789)
Supplement: Supplementary file 1 [file ijms-23-03789-s001.zip › ijms-1642826-supplementary.pdf]

New insights on hemopexin binding to Hemin and hemoglobin

Guilherme C. Lechuga[1,2] Paloma Napoleão-Pêgo[1], Carlos M. Morel[1], David W. Provance, Jr[1], Salvatore G. De-Simone[1,4,*]

– Supplementary Information –

Table S1: Linear mapping of hemopexin interaction with hemin and hemoglobin in the SPOT synthesis array (15 residues with overlapping of 10). Signal intensity of interaction regions of hemopexin sequences. Each peptide was identified by the Spot-synthesis membrane position numbering. Spot intensities below 50% were considered as background.

Spot	Peptide Sequence	Signal	Interaction sites	Molecule
A1	MARVLGAPVALGLWS	0		Hemin
A2	GAPVALGLWSLCWSL	0		Hemin
A3	LGLWSLCWSLAIATP	0		Hemin
A4	LCWSLAIATPLPPTS	0,54		Hemin
A5	AIATPLPPTSAHGNV	40,02		Hemin
A6	LPPTSAHGNVAEGET	6,65		Hemin
A7	AHGNVAEGETKPDPD	1,67		Hemin
A8	AEGETKPDPDVTERC	0		Hemin
A9	KPDPDVTERCSDGWS	0		Hemin
A10	VTERCSDGWSFDATT	0		Hemin
A11	SDGWSFDATTLDDNG	0		Hemin
A12	FDATTLDDNGTMLFF	2,35		Hemin
A13	LDDNGTMLFFKGEFV	0,09		Hemin
A14	TMLFFKGEFVWKSHK	35,08		Hemin
A15	KGEFVWKSHKWDREL	46,81		Hemin
A16	WKSHKWDRELISERW	26,83		Hemin
A17	WDRELISERWKNFPS	12,88		Hemin
A18	ISERWKNFPSPVDAA	2,36		Hemin
A19	KNFPSPVDAAFRQGH	87,53	KNFPSPVDAAFRQGH	Hemin
A20	PVDAAFRQGHNSVFL	28,92		Hemin
A21	FRQGHNSVFLIKGDK	59,85	FRQGHNSVFL	Hemin
A22	NSVFLIKGDKVWVYP	5,77		Hemin
A23	IKGDKVWVYPPEKKE	1,21		Hemin
A24	VWVYPPEKKEKGYPK	1,13		Hemin
B1	PEKKEKGYPKLLQDE	0,05		Hemin
B2	KGYPKLLQDEFPGIP	0,12		Hemin

B3	LLQDEFPGIPSPLDA	0,09		Hemin
B4	FPGIPSPLDAAVECH	2,35		Hemin
B5	SPLDAAVECHRGECQ	0,13		Hemin
B6	AVECHRGECQAEGVL	0,39		Hemin
B7	RGECQAEGVLFFQGD	0		Hemin
B8	AEGVLFFQGDREWFW	0		Hemin
B9	FFQGDREWFWDLATG	0		Hemin
B10	REWFWDLATGTMKER	0,34		Hemin
B11	DLATGTMKERSWPAV	0		Hemin
B12	TMKERSWPAVGNCSS	0		Hemin
B13	SWPAVGNCSSALRWL	6,61		Hemin
B14	GNCSSALRWLGRYYC	0,17		Hemin
B15	ALRWLGRYYCFQGNQ	1,72		Hemin
B16	GRYYCFQGNQFLRFD	1,79		Hemin
B17	FQGNQFLRFDPVRGE	14,92		Hemin
B18	FLRFDPVRGEVPPRY	8,19		Hemin
B19	PVRGEVPPRYPRDVR	6,09		Hemin
B20	VPPRYPRDVRDYFMP	2,56		Hemin
B21	PRDVRDYFMPCPGRG	6,9		Hemin
B22	DYFMPCPGRGHGHRN	36,9		Hemin
B23	CPGRGHGHRNGTGHG	28,47		Hemin
B24	HGHRNGTGHGNSTHH	27,56		Hemin
C1	GTGHGNSTHHGPEYM	7,9		Hemin
C2	NSTHHGPEYMRCSPH	32,91		Hemin
C3	GPEYMRCSPHLVLSA	23,89		Hemin
C4	RCSPHLVLSALTSDN	27,56		Hemin
C5	LVLSALTSDNHGATY	9,33		Hemin
C6	LTSDNHGATYAFSGT	2,27		Hemin
C7	HGATYAFSGTHYWRL	19,83		Hemin
C8	AFSGTHYWRLDTSRD	23,78		Hemin
C9	HYWRLDTSRDGWHSW	17,3		Hemin
C10	DTSRDGWHSWPIAHQ	48,07		Hemin
C11	GWHSWPIAHQWPQGP	51,32	GWHSWPIAHQ	Hemin
C12	PIAHQWPQGPSAVDA	2,45		Hemin
C13	WPQGPSAVDAAFSWE	3,79		Hemin
C14	SAVDAAFSWEEKLYL	0		Hemin
C15	AFSWEEKLYLVQGTQ	0		Hemin
C16	EKLYLVQGTQVYVFL	2,94		Hemin
C17	VQGTQVYVFLTKGGY	5,19		Hemin
C18	VYVFLTKGGYTLVSG	2,51		Hemin
C19	TKGGYTLVSGYPKRL	4,21		Hemin
C20	TLVSGYPKRLEKEVG	0,85		Hemin
C21	YPKRLEKEVGTPHGI	18,24		Hemin
C22	EKEVGTPHGIILDSV	23,41		Hemin
C23	TPHGIILDSVDAAFI	8,3		Hemin

C24	ILDSVDAAFICPGSS	0,05		Hemin
D1	DAAFICPGSSRLHIM	95,62		Hemin
D2	CPGSSRLHIMAGRRL	100	CPGSSRLHIMAGRRL	Hemin
D3	RLHIMAGRRLWWLDL	35,78		Hemin
D4	AGRRLWWLDLKSGAQ	12,88		Hemin
D5	WWLDLKSGAQATWTE	0,27		Hemin
D6	KSGAQATWTELPWPH	100	KSGAQATWTELPWPH	Hemin
D7	ATWTELPWPHEKVDG	34,34		Hemin
D8	LPWPHEKVDGALCME	0,51		Hemin
D9	EKVDGALCMEKSLGP	0		Hemin
D10	ALCMEKSLGPNSCSA	0,93		Hemin
D11	KSLGPNSCSANGPGL	2,89		Hemin
D12	NSCSANGPGLYLIHG	49,3	NSCSANGPGLYLIHG	Hemin
D13	NGPGLYLIHGPNLYC	20,55		Hemin
D14	YLIHGPNLYCYSDVE	0		Hemin
D15	PNLYCYSDVEKLNAA	0		Hemin
D16	YSDVEKLNAAKALPQ	8,86		Hemin
D17	KLNAAKALPQPQNVT	4,72		Hemin
D18	KALPQPQNVTSLLGC	0		Hemin
D19	LPQPQNVTSLLGCTH	67,03	LPQPQNVTSLLGCTH	Hemin
A1	MARVLGAPVALGLWS	97,21		Hemoglobin
A2	GAPVALGLWSLCWSL	81,13	MARVLGAPVALGLWSLCWSL	Hemoglobin
A3	LGLWSLCWSLAIATP	63,04		Hemoglobin
A4	LCWSLAIATPLPPTS	62,3		Hemoglobin
A5	AIATPLPPTSAHGNV	6,04		Hemoglobin
A6	LPPTSAHGNVAEGET	26,14		Hemoglobin
A7	AHGNVAEGETKPDPD	19,04		Hemoglobin
A8	AEGETKPDPDVTERC	6,26		Hemoglobin
A9	KPDPDVTERCSDGWS	11,88		Hemoglobin
A10	VTERCSDGWSFDATT	20,5		Hemoglobin
A11	SDGWSFDATTLDDNG	13,57		Hemoglobin
A12	FDATTLDDNGTMLFF	16,54		Hemoglobin
A13	LDDNGTMLFFKGEFV	4,53		Hemoglobin
A14	TMLFFKGEFVWKSHK	18,13		Hemoglobin
A15	KGEFVWKSHKWDREL	48,92		Hemoglobin
A16	WKSHKWDRELISERW	33,94		Hemoglobin
A17	WDRELISERWKNFPS	27,82		Hemoglobin
A18	ISERWKNFPSPVDAA	17,75		Hemoglobin
A19	KNFPSPVDAAFRQGH	15,78		Hemoglobin
A20	PVDAAFRQGHNSVFL	16,74		Hemoglobin
A21	FRQGHNSVFLIKGDK	16,11		Hemoglobin
A22	NSVFLIKGDKVWVYP	5,82		Hemoglobin
A23	IKGDKVWVYPPEKKE	8,37		Hemoglobin
A24	VWVYPPEKKEKGYPK	7,86		Hemoglobin
B1	PEKKEKGYPKLLQDE	38,22		Hemoglobin

B2	KGYPKLLQDEFPGIP	26,9		Hemoglobin
B3	LLQDEFPGIPSPLDA	48,4		Hemoglobin
B4	FPGIPSPLDAAVECH	39,86		Hemoglobin
B5	SPLDAAVECHRGECQ	25,56		Hemoglobin
B6	AVECHRGECQAEGVL	16,69		Hemoglobin
B7	RGECQAEGVLFFQGD	68,58		Hemoglobin
B8	AEGVLFFQGDREWFW	60,58		Hemoglobin
B9	FFQGDREWFWDLATG	79,47	FFQGDREWFWDLATG	Hemoglobin
B10	REWFWDLATGTMKER	61,36		Hemoglobin
B11	DLATGTMKERSWPAV	1,69		Hemoglobin
B12	TMKERSWPAVGNCSS	15		Hemoglobin
B13	SWPAVGNCSSALRWL	39,69		Hemoglobin
B14	GNCSSALRWLGRYYC	25,17		Hemoglobin
B15	ALRWLGRYYCFQGNQ	40,73		Hemoglobin
B16	GRYYCFQGNQFLRFD	36,62		Hemoglobin
B17	FQGNQFLRFDPVRGE	31,86		Hemoglobin
B18	FLRFDPVRGEVPPRY	23,55		Hemoglobin
B19	PVRGEVPPRYPRDVR	23,71		Hemoglobin
B20	VPPRYPRDVRDYFMP	29,98		Hemoglobin
B21	PRDVRDYFMPCPGRG	7,81		Hemoglobin
B22	DYFMPCPGRGHGHRN	16,15		Hemoglobin
B23	CPGRGHGHRNGTGHG	14,14		Hemoglobin
B24	HGHRNGTGHGNSTHH	11,4		Hemoglobin
C1	GTGHGNSTHHGPEYM	31,92		Hemoglobin
C2	NSTHHGPEYMRCSPH	40,59		Hemoglobin
C3	GPEYMRCSPHLVLSA	22,58		Hemoglobin
C4	RCSPHLVLSALTSDN	49,21		Hemoglobin
C5	LVLSALTSDNHGATY	74,61	LVLSALTSDNHGATYAFSGTHYWRLDTSRD	Hemoglobin
C6	LTSDNHGATYAFSGT	23,48		Hemoglobin
C7	HGATYAFSGTHYWRL	73,93		Hemoglobin
C8	AFSGTHYWRLDTSRD	79,03		Hemoglobin
C9	HYWRLDTSRDGWHSW	57,45		Hemoglobin
C10	DTSRDGWHSWPIAHQ	31,56		Hemoglobin
C11	GWHSWPIAHQWPQGP	17,75		Hemoglobin
C12	PIAHQWPQGPSAVDA	17,74		Hemoglobin
C13	WPQGPSAVDAAFSWE	30,88		Hemoglobin
C14	SAVDAAFSWEEKLYL	31,19		Hemoglobin
C15	AFSWEEKLYLVQGTQ	58,62	AFSWEEKLYLVQGTQ	Hemoglobin
C16	EKLYLVQGTQVYVFL	35,87		Hemoglobin
C17	VQGTQVYVFLTKGGY	26,6		Hemoglobin
C18	VYVFLTKGGYTLVSG	30,2		Hemoglobin
C19	TKGGYTLVSGYPKRL	33,21		Hemoglobin
C20	TLVSGYPKRLEKEVG	19,11		Hemoglobin
C21	YPKRLEKEVGTPHGI	18,27		Hemoglobin
C22	EKEVGTPHGIILDSV	8,29		Hemoglobin

C23	TPHGIILDSVDAAFI	24,75		Hemoglobin
C24	ILDSVDAAFICPGSS	0,77		Hemoglobin
D1	DAAFICPGSSRLHIM	62,57		Hemoglobin
D2	CPGSSRLHIMAGRRL	84,25		Hemoglobin
D3	RLHIMAGRRLWWLDL	100	RLHIMAGRRLWWLDLKSGAQATWTE	Hemoglobin
D4	AGRRLWWLDLKSGAQ	80,41		Hemoglobin
D5	WWLDLKSGAQATWTE	57,54		Hemoglobin
D6	KSGAQATWTELPWPH	51,04		Hemoglobin
D7	ATWTELPWPHEKVDG	43,29		Hemoglobin
D8	LPWPHEKVDGALCME	24		Hemoglobin
D9	EKVDGALCMEKSLGP	7,67		Hemoglobin
D10	ALCMEKSLGPNSCSA	5,99		Hemoglobin
D11	KSLGPNSCSANGPGL	16,15		Hemoglobin
D12	NSCSANGPGLYLIHG	29,43		Hemoglobin
D13	NGPGLYLIHGPNLYC	32,32		Hemoglobin
D14	YLIHGPNLYCYSDVE	34,96		Hemoglobin
D15	PNLYCYSDVEKLNAA	18,25		Hemoglobin
D16	YSDVEKLNAAKALPQ	6,32		Hemoglobin
D17	KLNAAKALPQPQNVT	31,52		Hemoglobin
D18	KALPQPQNVTSLLGC	3,79		Hemoglobin
D19	LPQPQNVTSLLGCTH	5,92		Hemoglobin
D20	DREKLQERLAKLAG	21,8		Hemoglobin
D21	QEVRKYFCV	3,06		Hemoglobin
D22	AVNFPNPPGKGGG	26,63		Hemoglobin
D23	AVNFPNPPGKGGG	24,13		Hemoglobin
D24	HPGSVNEFDF	23,57		Hemoglobin

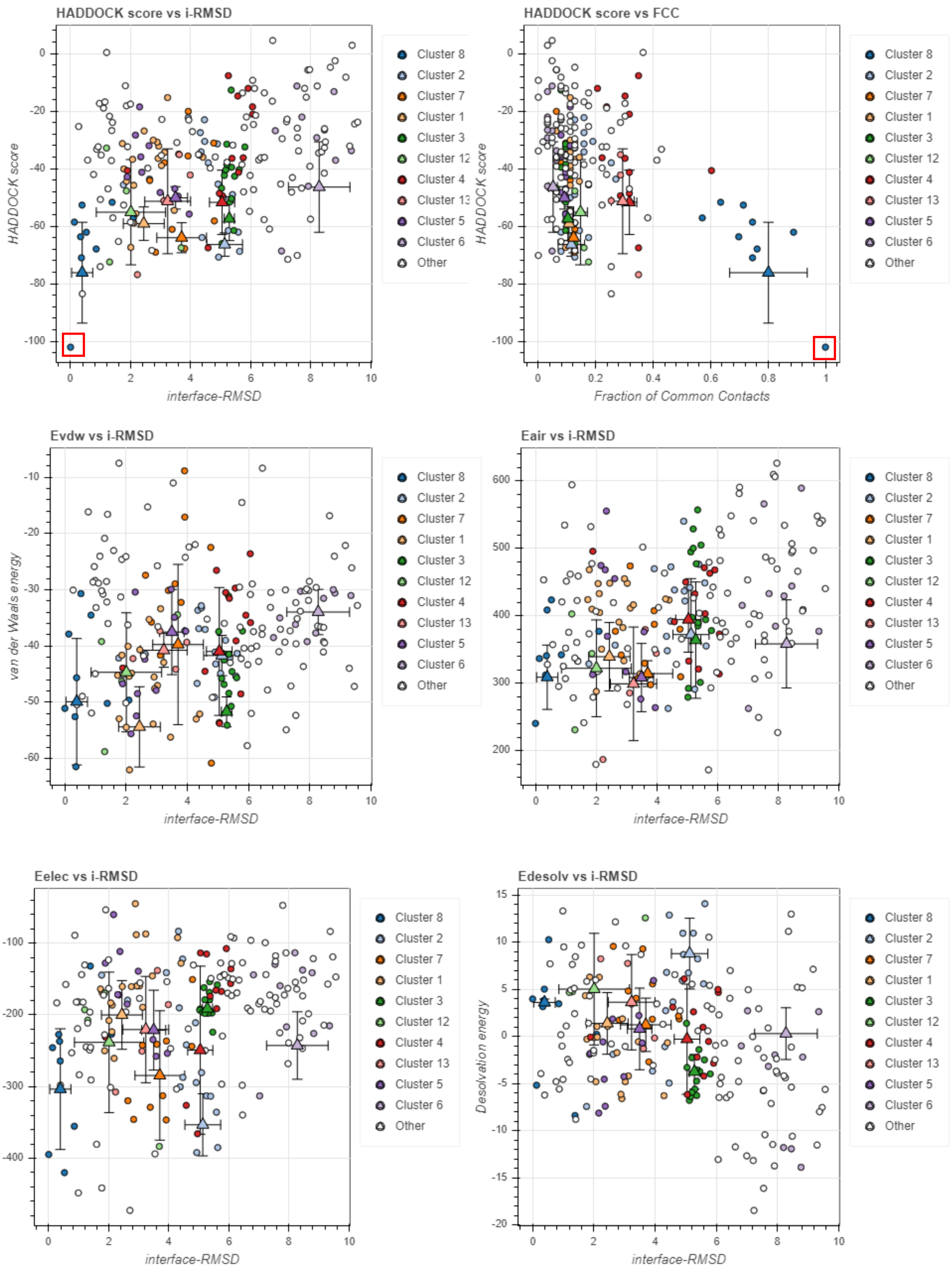

Figure S1: Interaction between hemopexin with hemoglobin. The simulation was performed by the server HADDOCK 2.4 (https://wenmr.science.uu.nl/haddock2.4/), generating 126 structures

represented by points in the graph presented in 13 clusters. Red squares represent the lowest energy structure.

Table S2: Biochemical and biophysical parameters and statistics of the 126 structures in 13 cluster(s), which represents 63 % of the water-refined models generated from HADDOCK webserver.

**Cluster 8**	
HADDOCK score	-76.1 +/- 15.2
Cluster size	9
RMSD from the overall lowest-energy structure	1.2 +/- 0.8
Van der Waals energy	-49.9 +/- 9.7
Electrostatic energy	-303.6 +/- 72.9
Desolvation energy	3.6 +/- 0.3
Restraints violation energy	308.7 +/- 41.3
Buried Surface Area	1742.0 +/- 50.1
Z-Score	-2.2
**Cluster 2**	
HADDOCK score	-66.4 +/- 3.5
Cluster size	17
RMSD from the overall lowest-energy structure	4.4 +/- 0.4
Van der Waals energy	-41.7 +/- 3.1
Electrostatic energy	-353.3 +/- 37.5
Desolvation energy	8.8 +/- 3.3
Restraints violation energy	372.0 +/- 71.8
Buried Surface Area	1705.8 +/- 49.1
Z-Score	-1.0
**Cluster 7**	
HADDOCK score	-64.0 +/- 4.5
Cluster size	10
RMSD from the overall lowest-energy structure	14.5 +/- 0.2
Van der Waals energy	-39.7 +/- 12.3
Electrostatic energy	-284.5 +/- 78.1
Desolvation energy	1.2 +/- 2.5
Restraints violation energy	314.1 +/- 16.6

Buried Surface Area	1474.7 +/- 65.6
Z-Score	-0.7
**Cluster 1**	
HADDOCK score	-59.1 +/- 5.0
Cluster size	19
RMSD from the overall lowest-energy structure	14.0 +/- 0.4
Van der Waals energy	-54.4 +/- 6.2
Electrostatic energy	-200.1 +/- 41.5
Desolvation energy	1.4 +/- 2.8
Restraints violation energy	339.2 +/- 43.9
Buried Surface Area	1409.2 +/- 51.1
Z-Score	-0.2
**Cluster 3**	
HADDOCK score	-57.4 +/- 6.6
Cluster size	14
RMSD from the overall lowest-energy structure	13.7 +/- 0.5
Van der Waals energy	-51.7 +/- 2.3
Electrostatic energy	-191.4 +/- 7.8
Desolvation energy	-3.8 +/- 1.6
Restraints violation energy	363.8 +/- 74.6
Buried Surface Area	1655.9 +/- 48.3
Z-Score	0.0
**Cluster 12**	
HADDOCK score	-55.1 +/- 15.8
Cluster size	4
RMSD from the overall lowest-energy structure	10.3 +/- 0.6
Van der Waals energy	-44.7 +/- 9.2
Electrostatic energy	-238.3 +/- 84.8
Desolvation energy	5.0 +/- 5.1
Restraints violation energy	322.0 +/- 62.1
Buried Surface Area	1466.5 +/- 143.4
Z-Score	0.3
**Cluster 4**	
HADDOCK score	-51.7 +/- 9.7
Cluster size	12
RMSD from the overall lowest-energy structure	6.1 +/- 0.2
Van der Waals energy	-41.0 +/- 9.8

Electrostatic energy	-249.3 +/- 101.5
Desolvation energy	-0.3 +/- 5.1
Restraints violation energy	394.1 +/- 41.8
Buried Surface Area	1369.4 +/- 73.0
Z-Score	0.7
**Cluster 13**	
HADDOCK score	-51.3 +/- 15.8
Cluster size	4
RMSD from the overall lowest-energy structure	6.8 +/- 0.7
Van der Waals energy	-40.8 +/- 2.6
Electrostatic energy	-220.5 +/- 64.2
Desolvation energy	3.6 +/- 4.4
Restraints violation energy	299.0 +/- 72.9
Buried Surface Area	1385.0 +/- 88.9
Z-Score	0.8
**Cluster 5**	
HADDOCK score	-50.1 +/- 3.4
Cluster size	10
RMSD from the overall lowest-energy structure	15.0 +/- 0.3
Van der Waals energy	-37.5 +/- 6.6
Electrostatic energy	-221.3 +/- 48.2
Desolvation energy	0.8 +/- 3.8
Restraints violation energy	308.3 +/- 43.8
Buried Surface Area	1535.2 +/- 128.9
Z-Score	0.9
**Cluster 6**	
HADDOCK score	-46.4 +/- 13.6
Cluster size	10
RMSD from the overall lowest-energy structure	8.1 +/- 0.8
Van der Waals energy	-34.0 +/- 3.5
Electrostatic energy	-242.8 +/- 40.8
Desolvation energy	0.3 +/- 2.4
Restraints violation energy	358.2 +/- 56.5
Buried Surface Area	1278.2 +/- 61.4
Z-Score	1.3

Table S3: Prediction of Hot spot interactions between Hemopexin and Hemoglobin β-chain using bioinformatics (https://mitchell-web.ornl.gov/KFC_Server/).

			KFC2-A	KFC2-A
Chain	Res	Num	Class	Conf
A - Hemopexin	TRP	314	-------	-2.29
A - Hemopexin	HIS	353	-------	-0.36
A - Hemopexin	PRO	367	-------	-2.50
A - Hemopexin	GLY	368	-------	-2.05
A - Hemopexin	SER	369	-------	-1.44
A - Hemopexin	**SER**	**370**	**Hotspot**	0.12
A - Hemopexin	**ARG**	**371**	**Hotspot**	**0.66**
A - Hemopexin	**TRP**	**382**	**Hotspot**	**1.09**
A - Hemopexin	LEU	383	-------	-0.69
A - Hemopexin	**ASP**	**384**	**Hotspot**	0.91
A - Hemopexin	LYS	386	-------	-0.86
A - Hemopexin	SER	387	-------	-1.10
A - Hemopexin	ALA	389	-------	-1.92
A - Hemopexin	GLN	390	-------	-1.69
A - Hemopexin	ALA	391	-------	-1.35
A - Hemopexin	**THR**	**392**	**Hotspot**	1.24
A - Hemopexin	TRP	393	-------	-0.28
A - Hemopexin	**THR**	**394**	**Hotspot**	1.26
A - Hemopexin	GLU	395	-------	-1.63
A - Hemopexin	LEU	396	-------	-0.01
A - Hemopexin	PRO	397	-------	-1.54
A - Hemopexin	GLU	440	-------	-1.66
A - Hemopexin	ASN	443	-------	-1.05
A - Hemopexin	ALA	444	-------	-2.44
B - Hemoglobin	TRP	15	-------	-2.93
B - Hemoglobin	ASP	21	-------	-2.95
B - Hemoglobin	SER	44	-------	-1.71
B - Hemoglobin	PRO	58	-------	-2.23
B - Hemoglobin	LYS	59	-------	-0.35
B - Hemoglobin	ALA	62	-------	-1.30
B - Hemoglobin	HIS	63	-------	-1.80
B - Hemoglobin	**LYS**	**65**	**Hotspot**	**0.35**
B - Hemoglobin	**LYS**	**66**	**Hotspot**	**1.77**
B - Hemoglobin	GLY	69	-------	-0.02
B - Hemoglobin	ALA	70	-------	-1.75
B - Hemoglobin	SER	72	-------	-0.88
B - Hemoglobin	**ASP**	**73**	**Hotspot**	0.22
B - Hemoglobin	ALA	76	-------	-1.52
B - Hemoglobin	HIS	77	-------	-1.69

B - Hemoglobin	GLY	83	-------	-2.21
B - Hemoglobin	**THR**	**84**	**Hotspot**	0.27
B - Hemoglobin	**THR**	**87**	**Hotspot**	0.06
B - Hemoglobin	**LEU**	**88**	**Hotspot**	**0.93**
B - Hemoglobin	GLU	90	-------	-1.39
B - Hemoglobin	LEU	91	-------	-0.64
B - Hemoglobin	LYS	95	-------	-1.14
B - Hemoglobin	LEU	96	-------	-2.46

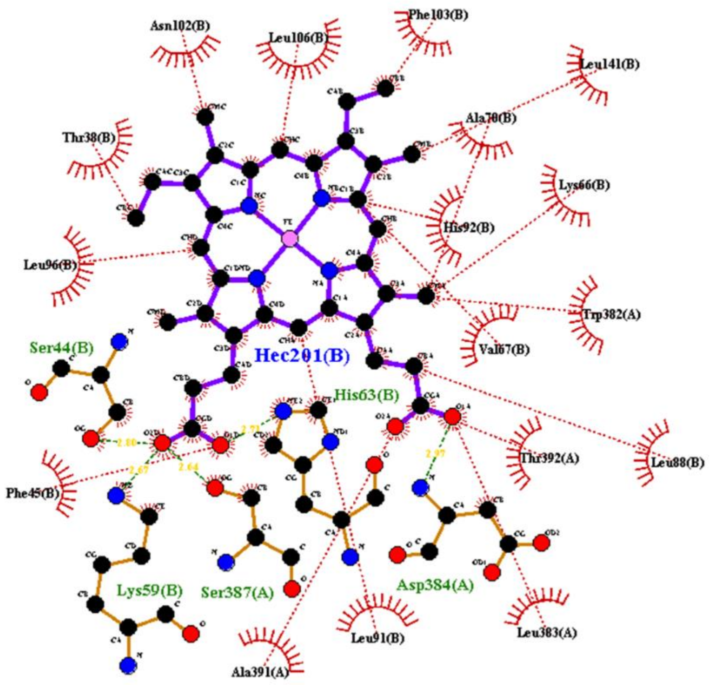

Figure S2: Interaction of residues with heme group inside hemoglobin β-chain and hemopexin in protein-protein interface. Residues interacted with heme (Purple) by Hydrophobic interactions that are represented by red spokes radiating towards the ligand atoms they contact, and hydrogen bonds are showed in green dashes with distances (Å) in yellow. Hydrogen bonds and hydrophobic interactions shown in this illustration are calculated through the Ligplot algorithm.
